# 2060. Evaluation of the real world incidence of integrase inhibitor resistance since adoption as guideline preferred therapy

**DOI:** 10.1093/ofid/ofac492.1682

**Published:** 2022-12-15

**Authors:** Jenna Januszka, Emily N Drwiega, Emily N Drwiega, Rodrigo M Burgos, Sarah M Michienzi, Renata Smith, Melissa E Badowski

**Affiliations:** University of Illinois at Chicago, Chicago, Illinois; University of Illinois Chicago, Chicago, Illinois; University of Illinois Chicago, Chicago, Illinois; University of Illinois at Chicago, Chicago, Illinois; University of Illinois Chicago College of Pharmacy, Chicago, Illinois; University of Illinois at Chicago, Chicago, Illinois; University of Illinois Chicago, Chicago, Illinois

## Abstract

**Background:**

There are limited data reporting real-world incidence of integrase inhibitor resistance (INSTI-R) since the approval and first-line treatment recommendation of INSTIs in the US. A recent analysis of the national surveillance data estimated INSTI-R to be 6.3%. The purpose of this study was to describe real-world incidence of INSTI-R in patients who used an INSTI-based single tablet regimen (STR) in a major metropolitan area and identify risk factors for resistance.

**Methods:**

This was a retrospective study of adult patients living with human immunodeficiency virus (HIV) who were prescribed an INSTI-STR between September 2017 and September 2020 and followed for > 12 months at the University of Illinois Chicago Community Clinic Network (UCCN). The primary endpoint was the difference in INSTI-R in UCCN patients compared to the national prevalence of 6.3%. Other outcomes included development of virologic failure (VF), defined as 2 consecutive HIV-1 viral loads (VL) > 200 copies/mL after week 24, and documented INSTI-R mutations. Patient specific factors associated with medication nonadherence were also collected. All endpoints were analyzed using chi-square and Fisher’s exact tests.

**Results:**

Of 948 patients screened, 248 patients were included. Baseline characteristics are summarized in Table 1. VF occurred in 17 subjects (6.8%). Two of the 17 (11.8%) received subsequent INSTI-R testing and nine eventually virally suppressed without regimen changes (52.9%). Patients without VF were assumed to have no INSTI-R.

No subjects developed INSTI-R, which was significantly less than the national prevalence of 6.3% (p=0.0029). Patients with a high VL at baseline were more likely to experience VF (p=0.001). The most common factors associated with nonadherence in patients with VF are summarized in Table 2.

**Figure:**
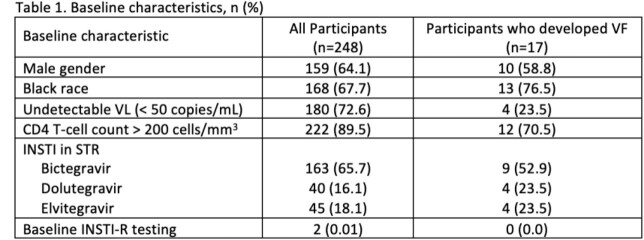

**Figure:**
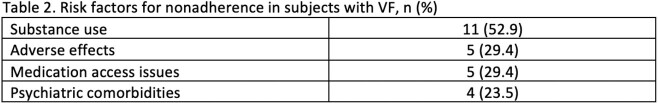

**Conclusion:**

The true rate of INSTI-R in UCCN patients is still unknown and factors associated with developing INSTI-R were unable to be assessed. Among patients at UCCN on INSTI-based STRs, INSTI-R rates were lower than the national average. Future analyses should also include patients on INSTI-based non-STR regimens as increased pill burden is a known risk factor for nonadherence leading to VF and drug resistance.

**Disclosures:**

**All Authors**: No reported disclosures.

